# Invasive *Acacia mangium* Leaf Litter Modifies Soil Chemical Properties of A Bornean Tropical Heath Forest: A Soil Incubation Study

**DOI:** 10.21315/tlsr2025.36.1.14

**Published:** 2025-03-30

**Authors:** Mohamad Hilmi Ibrahim, Faizah Metali, Kushan U Tennakoon, Rahayu Sukmaria Sukri

**Affiliations:** 1 Institute for Biodiversity and Environmental Research, Universiti Brunei Darussalam, Jalan Tungku Link, Gadong BE1410, Brunei Darussalam; 2 Agrotechnology Programme, Faculty of Resource Science and Technology, Universiti Malaysia Sarawak, 94300 Kota Samarahan, Sarawak, Malaysia; 3 Environmental and Life Sciences Programme, Faculty of Science, Universiti Brunei Darussalam, Jalan Tungku Link, Gadong BE1410, Brunei Darussalam; 4 Institute of Innovation, Science and Sustainability, Federation, University Australia, Berwick Campus, No.100 Clyde Road, Berwick, VIC 3806, Australia

**Keywords:** Brunei Darussalam, Bornean Forests, Exotic Species, Non-native Species, Soil Chemistry, Brunei Darussalam, Hutan Borneo, Spesies Eksotik, Spesies Bukan Asli, Kimia Tanah

## Abstract

This study investigated the effects of *Acacia mangium* Willd. leaf litter on soil chemical properties of a tropical heath forest in Borneo using a controlled soil incubation experiment. The litter of exotic *A. mangium* and selected native heath forest species (*Buchanania arborescens* Blume., *Calophyllum inophyllum* L., *Dillenia suffruticosa* Griff. and *Ploiarium alternifolium* Vahl.) were incubated with heath forest soils collected under natural conditions and nine different treatments of heath forest soils (soils without leaf litter, soils treated with single species leaf litter, and soils treated with native leaf litter with and without *A. mangium* leaf litter). We quantified mass litter loss (%), and soil concentrations of exchangeable nitrogen ( ${\text{NO}}_{3}^{-}$ and ${\text{NH}}_{4}^{+}$) and cations (K*^+^*, Ca^2+^ and Mg^2+^), available phosphorus (P), total organic carbon (TOC) and organic matter (OM), and total acidity with exchangeable concentrations of Al^3+^ and H^+^ in each treatment after a 9-month incubation period. Mass litter loss (%) varied significantly between species, with *A. mangium* leaf litter only showing higher mass loss than *D. suffruticosa* litter, but lower than *C. inophyllum* litter. The effects of incubation with single-species native leaf litter were variable and species-specific, but incubation with *A. mangium* litter increased soil pH and exchangeable ${\text{NO}}_{3}^{-}$ and K^+^ concentrations and decreased exchangeable Al^3+^ concentrations. Soils incubated with a combination of *C. inophyllum* and *A. mangium* leaf litters, as opposed to those incubated with *C. inophyllum* alone, exhibited decreased pH, lower total organic carbon (TOC), and reduced exchangeable concentrations of potassium (K^+^) and magnesium (Mg^2+^). Additionally, there was an increase in organic matter (OM) content, total acidity, and exchangeable concentrations of ammonium ( ${\text{NH}}_{4}^{+}$) and hydrogen (H^+^). Our results provide preliminary evidence that *C. inophyllum* may be a promising native plant species for use in enrichment planting of degraded or disturbed tropical heath forests with co-occurring invasive *A. mangium*.

## Abstract (fr)

**Abstrak:**

Kajian ini menyiasat kesan daun reput *Acacia mangium* Willd. terhadap sifat kimia tanah hutan kerangas tropika di Borneo menggunakan pendekatan eksperimen inkubasi tanah terkawal. Daun reput spesies asing *A. mangium* dan spesies hutan kerangas asli terpilih (*Buchanania arborescens* Blume., *Calophyllum inophyllum* L., *Dillenia suffruticosa* Griff. dan *Ploiarium alternifolium* Vahl.) diinkubasi dengan tanah hutan kerangas yang dikumpul di bawah keadaan semula jadi dengan sembilan rawatan tanah hutan kerangas yang berbeza (tanah tanpa daun reput, tanah yang dirawat dengan daun reput spesies tunggal dan tanah yang dirawat dengan daun reput asli dengan dan tanpa daun reput *A. mangium*). Kemudian, kami mengukur kehilangan jisim daun reput (%), dan kepekatan tanah nitrogen boleh tukar ( ${\text{NO}}_{3}^{-}$ dan ${\text{NH}}_{4}^{+}$) dan kation (K^+^, Ca^2+^ dan Mg^2+^), fosforus tersedia (P), jumlah karbon organik (TOC) dan bahan organik (OM), dan jumlah keasidan dengan kepekatan boleh tukar Al^3+^ dan H^+^ dalam setiap rawatan selepas tempoh inkubasi 9 bulan. Kehilangan jisim daun reput (%) berbeza dengan ketara di antara spesies, dengan daun reput *A. mangium* sahaja menunjukkan kehilangan jisim yang lebih tinggi daripada daun reput *D. suffruticosa*, tetapi lebih rendah daripada daun reput *C. inophyllum*. Kesan inkubasi dengan daun reput asli spesies tunggal adalah berubah-ubah dan spesifik kepada spesies, tetapi inkubasi dengan daun reput *A. mangium* meningkatkan pH tanah dan kepekatan ${\text{NO}}_{3}^{-}$ dan K^+^ boleh tukar dan mengurangkan kepekatan Al^3+^ boleh tukar. Tanah yang diinkubasi dengan kombinasi daun reput *C. inophyllum* dan *A. mangium*, berbanding dengan *C. inophyllum* sahaja, menunjukkan penurunan pH, jumlah karbon organik (TOC) yang lebih rendah, dan kepekatan boleh tukar kalium (K^+^) dan magnesium (Mg^2+)^ yang berkurangan. Selain itu, terdapat peningkatan dalam kandungan bahan organik (OM), keasidan total, dan kepekatan boleh tukar ammonium ( ${\text{NH}}_{4}^{+}$) dan hidrogen (H^+^). Hasil dapatan kami memberikan bukti awal bahawa *C. inophyllum* mungkin menjadi spesies tanaman asli yang berpotensi untuk digunakan dalam pengayaan penanaman hutan kerangas tropika yang terdegradasi atau terganggu dengan kehadiran spesies invasif *A. mangium* pada masa yang sama.

**Highlights**

- *Acacia mangium* litter significantly increased soil pH and exchangeable NO_3_^−^ and K^+^ concentrations while reducing exchangeable Al^3+^ concentrations in tropical heath forest soils.
- Co-incubation of heath forest soils with invasive *A. mangium* and native *Calophyllum inophyllum* litter enhanced organic matter content and reduced total acidity, demonstrating its potential for enrichment planting in degraded tropical heath forests.
- Negative impact on tropical heath forest soils through continuous exposure of to *A. mangium* leaf litter can potentially be mediated through mixed species incubation with native heath forest species litter.

## INTRODUCTION

Alien invasive tree species are considered a threat to biodiversity in a number of countries, including Southeast Asia (Fuentes-Ramírez *et al*. 2011; Le Maitre *et al*. 2011; Corlett 2010; Peh 2010). *Acacia*, a genus of trees from the family Fabaceae, is a well-documented invasive species in Borneo (Ibrahim *et al*. 2023; Richardson & Rejmánek 2011). In Brunei Darussalam, located in northwestern Borneo, three *Acacia* species (*Acacia mangium* Willd., *Acacia cincinnata* F. Muell, and *Acacia auriculiformis* Benth.) were first introduced in the mid-1990s for plantation forestry and restoration of degraded forests (Osunkoya *et al*. 2005). As nitrogen-fixing fast-growing plants, these exotic *Acacia* species have been able to outcompete co-occurring native species particularly in nutrient-poor soils (Osunkoya *et al*. 2005). Following their introduction, *Acacia* invasion is now recorded in Brunei’s urban forests and coastal forests, and in disturbed tropical Bornean heath and mixed dipterocarp forests (Jaafar *et al*. 2022a; Jambul *et al*. 2020; Tuah *et al*. 2020; Suhaili 2017; Din *et al*. 2015). These invasive *Acacia* species have negatively affected ecosystem processes, particularly soil properties, nutrient cycling processes and litter decomposition (Ibrahim *et al*. 2023; Jaafar *et al*. 2022b).

Leaf litter affects soil chemical properties through litter decomposition, which is in turn influenced by environmental conditions including both ambient air and soil temperatures, as well as the moisture levels present in the air and within the soil itself, the presence of microorganisms particularly microbial and fungal populations, and litter quality (Birouste *et al*. 2012; Bakker *et al*. 2011; Lützow *et al*. 2006). Leaf litter decomposition releases a flux of carbon, followed by organic matter, and finally soil humus enriched with basic cations such as potassium, calcium and magnesium (Jeyanny *et al*. 2015). However, the presence of alien invasive plants such as *Acacia* can interfere with litter decomposition processes in native habitat soils. Xiang and Bauhus (2007) recorded increased decomposition rates of naturally occurring *Eucalyptus globulus* leaf litter in the presence of exotic *Acacia mearnsii* in Victoria, Australia because of elevated N concentrations in soils. In contrast, Castro-Díez *et al*. (2012) reported that leaf litter from exotic *Eucalyptus globulus* and *Acacia dealbata* in Spain were less decomposed than litter from native tree species *Quercus robur* and *Pinus pinaster*. In Congo, Bernhard-Reversat and Schwartz (2002) reported that the decomposition rate of *Acacia* species leaf litter was intermediate between a *Eucalypt* plantation and a natural forest.

Comparisons of decomposition processes between invasive and native plants typically utilise the litter bag technique (Berg & Laskowski 2005). Using this technique, Le Maitre *et al*. (1996) studied the nitrogen cycling of invasive *Acacia* species that have spread through the fynbos biome in South Africa. In a separate study, Richardson and Rejmánek (2011) examined the effects of litter from *Acacia* species on the soil chemistry in Mediterranean-type ecosystems. In tropical Brunei Darussalam, Suhaili (2017) reported higher decomposition rates and nutrient release by *A. mangium* phyllodes compared to leaf litter from mixed native heath forests while Jaafar *et al*. (2022b) reported leaf litter decomposition rates and nutrient release were lower in *Acacia*-invaded mixed dipterocarp forest (AMDF) than in the *Acacia*-invaded heath forest (AHF), lowland heath (HF) and mixed dipterocarp forests (MDF).

Although litter decomposition studies using the litter bag have shown that invasive species litter decomposes at different rates than native litter, studies that directly evaluate the effects of invasive species litter on soil properties of invaded habitats remain limited. The soil incubation method (see Castro-Díez *et al*. 2012) was developed as an experimental approach in a controlled environment to quantify the direct effects of invasive litter decomposition on native soils. We therefore utilised the soil incubation method to investigate the contrasting effects of leaf litter decomposition from invasive *A. mangium* and selected native plant species on the chemical properties of tropical heath forest soils. We formulated two research questions:

1. Are there differences in the chemical properties of soils in tropical heath forests when incubated with leaf litter of a single species (*A. mangium* and a native species) and without litter?
2. Are there differences in chemical properties of tropical heath forest soils when incubated with native species leaf litter (mixture) in the presence or absence of invasive *A. mangium* leaf litter?

## MATERIALS AND METHODS

### Litter And Soil Collection

We collected freshly fallen leaf litter from three native tropical heath species (*Buchanania arborescens* (Blume) Blume, *Calophyllum inophyllum* L. and *Ploiarium alternifolium* (Vahl) Melch), a common pioneer species (*Dillenia suffruticosa* (Griff.) Martelli) and freshly fallen phyllodes (hereafter leaf litter) of the invasive *A. mangium* Willd. These four native species were selected because they are the most abundant native species in the tropical coastal heath forests in Brunei Darussalam near the Universiti Brunei Darussalam campus (Tuah *et al*. 2020). Leaf litter of *B. arborescens*, *C. inophyllum*, *D. suffruticosa* and *P. alternifolium* were collected from heath forests that were not infested with *Acacia* (N 04°57.698 E 114°52.307 a.s.l.), while leaf litter of *A. mangium* was collected from *Acacia-*invaded heath forests (N 04°57.388 E 114°52.194 a.s.l.). The collected samples were cleaned of all residues with tissue paper and air-dried for two days, and air-dried leaf litter was cut into small pieces (about 1.5 cm^2^) with scissors. A detailed description of the study sites is included in Ibrahim *et al*. (2023).

We also collected fresh leaves of the five study species to quantify foliar nutrient composition before decomposition (Table 1). Three different trees (about 5 m high) of each species were selected (with distances of more than 3 m between sampled trees) and one branch with healthy and mature leaves was taken from each tree with pruning shears. The branches were taken to the laboratory for nutrient analysis (total N, P, Ca, K and Mg) as described by Jaafar *et al*. (2022b). In addition, 50 kg of soil were collected at a depth of 0 cm to 25 cm in an intact, non-invaded heath forest (N 04°57.698 E 114°52.307). The soil samples were air-dried for 6 days, crushed with a pestle and mortar, and passed through a 2-mm sieve following Ibrahim *et al*. (2023).

### Soil Incubation Experiment

The soil incubation experiment was conducted in a 6 m × 4 m closed room at a relative humidity of 75.2 ± 2.3% and a room temperature of 26.1 ± 1.7°C. Sixty soil columns made of polyethylene plastic bottles (17 cm diameter, 6 cm height) were drilled with 26 holes (3 mm diameter) at the bottom and covered with Whatman filter paper No. 2 (Dise & Wright 1995; Castro-Díez *et al*. 2012). Soil bulk density (Brady & Weil 2002) at the intact heath forest site was first quantified, and the value was used to estimate the quantity of soil (i.e., soil without water content) to be used in each column. This resulted in each empty soil column being filled with 1.3 kg of air-dried soil, to simulate the natural condition of the heath soil at the study sites. The litter square technique described by Todd *et al*. (2000) was used to determine the required litter quantity in each soil column. Approximately 92 g of litter could be accommodated in a square (30 cm × 30 cm), resulting in approximately 24 g of leaf litter added to the air-dried soil in each soil column in a polyethylene bottle.

The soil incubation experiment was set up as a completely randomised design (CRD) consisting of 10 treatments with different combinations of study types, with 3 replicates per treatment (Table 2). Treatment 0 represented the control (soil only, without litter), while treatments 1 to 5 included litter from a single species. Treatments 6 to 9 were incubation treatments with litter from *A. mangium* and a native species (*B. arborescens, C. inophyllum, D. suffruticosa* and *P. alternifolium*) to simulate the effects of *A. mangium* invasion in a Bornean tropical heath forest.

All polyethylene columns received 1 L (100% field capacity) of distilled water at the start of the experiment in April 2015, followed by 100 mL of distilled water every week until December 2015. These water volumes were determined based on average yearly rainfall divided by the number of rainfall occurrences in a year for the study sites using the Brunei Meteorological Department database in 2015 (Brunei Meteorological Department, unpublished data). The soil incubation experiment was conducted over 9 months (Castro-Díez *et al*. 2012).

### Litter and Soil Properties Analysis

At the end of the 9-month incubation period, all remaining leaf litter samples were collected and weighed to determine the percentage mass litter loss using the formula:


$$\%\;\text{litter loss}=\frac{\text{Mass of leaf litter at the start }(\text{g})-\text{Mass of leaf litter remaining }(\text{g})}{\text{Mass of leaf litter at the start }(\text{g})}\times 100$$

For the soils, samples were taken from each column and prepared for the respective soil chemical analyses. Soil samples were air-dried at the end of the 9-month incubation period, passed through a 2-mm sieve, and ground with a pestle and mortar. The total organic carbon (TOC) and organic matter (OM) content of all remaining leaf litter samples was determined using the dry combustion method (Cheftetz *et al*. 1996). Soil pH in water and KCl (pH_water_ and pH_KCl_) were determined according to the methods of Tan (2005), while exchangeable potassium (K^+^), calcium (Ca^2+^) and magnesium (Mg^2+^) were extracted according to the methods of

Allen *et al*. (1989). Total nutrient contents (K^+^, Ca^2+^ and Mg^2+^) for soil and leaf litter samples were determined using a flame atomic absorption spectrophotometer (AAS; iCE 300, Thermo Fisher Scientific^®^, NSW, Australia). Potassium chloride (KCl) solution was used to extract exchangeable ammonium ( ${\text{NH}}_{4}^{+}$) and nitrate ( ${\text{NO}}_{3}^{-}$) from the soil and both nutrients in the samples were quantified using a Flow Injection Analyzer (FIAstarTM5000, FOSS^®^, Hoganas, Sweden) (Keeney & Nelson 1982). Available phosphorus was determined according to Allen *et al*. (1989) using a UV-VIS spectrometer (UV1800, Shimadzu, Kyoto, Japan) at a wavelength of 880 nm after the samples had been extracted with a Bray solution (0.03 N ammonium fluoride in 0.025 N HCI). To determine the total acidity and exchangeable concentrations of aluminium (Al^3+^) and hydrogen (H^+^), soil samples were extracted with 1 M KCl, and the extracts were then titrated with 0.01 M NaOH and 0.01 M HCl as described by Rowell (1994).

### Statistical Analysis

Between-species differences in soil properties (pH, ${\text{NO}}_{3}^{-}$ and ${\text{NH}}_{4}^{+}$, K*^+^*, Ca^2+^, Mg^2+^, available P, TOC, OM, total acidity, Al^3+^ and H^+^) after incubation with leaf litter of native heath forest species (T2 to T5), invasive *A. mangium* litter (T1) and the control treatment (T0) were investigated using a one-way analysis of variance (ANOVA), followed by Tukey’s HSD tests. Unpaired *t*-tests were used to determine the differences in soil chemical properties when incubated with native species litter in the presence (T6 to T9) or absence (T2 to T5) of *A. mangium* leaf litter. For all one-way ANOVA and *t*-tests, assumptions of normality were tested using Shapiro-Wilk test and assumptions of homogeneity of variance were tested using Levene’s test, and neither assumptions were violated. All analyses were performed using Statistical Analysis System version 9.2 (SAS ver.9.2).

## RESULTS

### Variations of Percentage Mass Litter Loss and Selected Soil Chemical Properties when Incubated with Litter from a Single Species

The percentage mass litter loss of *A. mangium* and three of the native species (*C. inophyllum, D. suffruticosa* and *P. alternifolium*) varied significantly (*p* < 0.05; Table 3). The highest percentage mass litter loss recorded by *C. inophyllum* litter with 27.89%, while the lowest percentage mass litter loss recorded was for *D. suffruticosa* litter with 2.20% (Table 3). Percentage mass litter loss for *A. mangium litter* was significantly higher than percentage mass litter loss for *D. suffruticosa* litter, and significantly lower than percentage mass litter loss for *C. inophyllum* litter but did not significantly differ from percentage mass litter loss for *B. arborescens* and *P. alternifolium* (Table 3).

Several soil chemical properties (pH in KCl, pH in water, exchangeable concentrations of ${\text{NO}}_{3}^{-}$, Al^3+^, H^+^, K^+^, Ca^2+^ and Mg^2+^, and total acidity) recorded significant differences between the control treatment (soil samples incubated in a column with no litter) and treatments containing litter from a single-species: *Acacia mangium* litter and heath forest species litter (*B. arborescens, C. inophyllum, D. suffruticosa* and *P. alternifolium*), though these significant differences were variable and did not follow a consistent pattern (Table 3). However, TOC, OM, exchangeable ${\text{NH}}_{4}^{+}$ and available P concentrations showed no significant mean differences among the treatments, hence they were excluded from this table.

Soil pH in water and KCl (pH_water_ and pH_KCl_) showed significant differences after incubation with litter (Table 3). Soil samples that were incubated without any litter recorded significantly lower pH_KCl_ (3.65) compared to soil samples incubated with *C. inophyllum* litter (3.75) but did not show significant differences with other single litter treatments, including that of invasive *A. mangium* leaf litter treatment. Soil samples that were incubated without any litter recorded significantly lower pH_water_ (4.61) compared to soil samples incubated with *A. mangium* litter (4.94), *B. arborescens* litter (4.94) and *C. inophyllum* litter (4.83) but did not show significant differences with other single litter treatments (Table 3).

Total acidity was significantly lower in soil samples incubated with *A. mangium* litter (2.88 mg kg^−1^), *C. inophyllum* litter (2.67 mg kg^−1^) and *P. alternifolium* litter (2.91 mg kg^−1^) than the samples incubated without any litter (3.24 mg kg^−1^). In contrast, exchangeable Al^3+^ concentration was significantly higher in soil samples incubated without any litter (2.55 mg kg^−1^) than soil samples incubated with tree litter (Table 3). For exchangeable H^+^, those soil samples incubated without any litter (T0) recorded significantly lower exchangeable H^+^ when compared to soil samples incubated with *P. alternifolium* litter but did not show significant differences with other single litter treatments, including that of invasive *A. mangium* litter (Table 3).

Soil exchangeable nitrate ( ${\text{NO}}_{3}^{-}$) concentration of soil samples incubated without any litter was significantly lower when compared to those of *A. mangium* litter, *C. inophyllum* litter and *P. alternifolium* litter but did not significantly differ with *B. arborescens* litter (Table 3). Exchangeable cation concentrations showed significant differences after the incubation experiment (Table 3). Those soil samples incubated with tree litter recorded significantly higher exchangeable K^+^ concentration when compared to those soil samples incubated without any litter (Table 3). Exchangeable Mg^2+^ concentrations of soil samples incubated with no litter was significantly lower when compared to *C. inophyllum* litter, *D. suffruticosa* litter and *P. alternifolium* litter (Table 3). Exchangeable Ca^2+^ concentration was significantly higher in soil samples incubated with *P. alternifolium* litter (0.073 mg kg^−1^) than those soil samples incubated without any litter and other single litter treatments (Table 3).

### Variations of Soil Chemical Properties After Incubation with Tropical Heath Forest Leaf Litter in the Presence or Absence of *A. mangium* Leaf Litter

**Figure 1:**
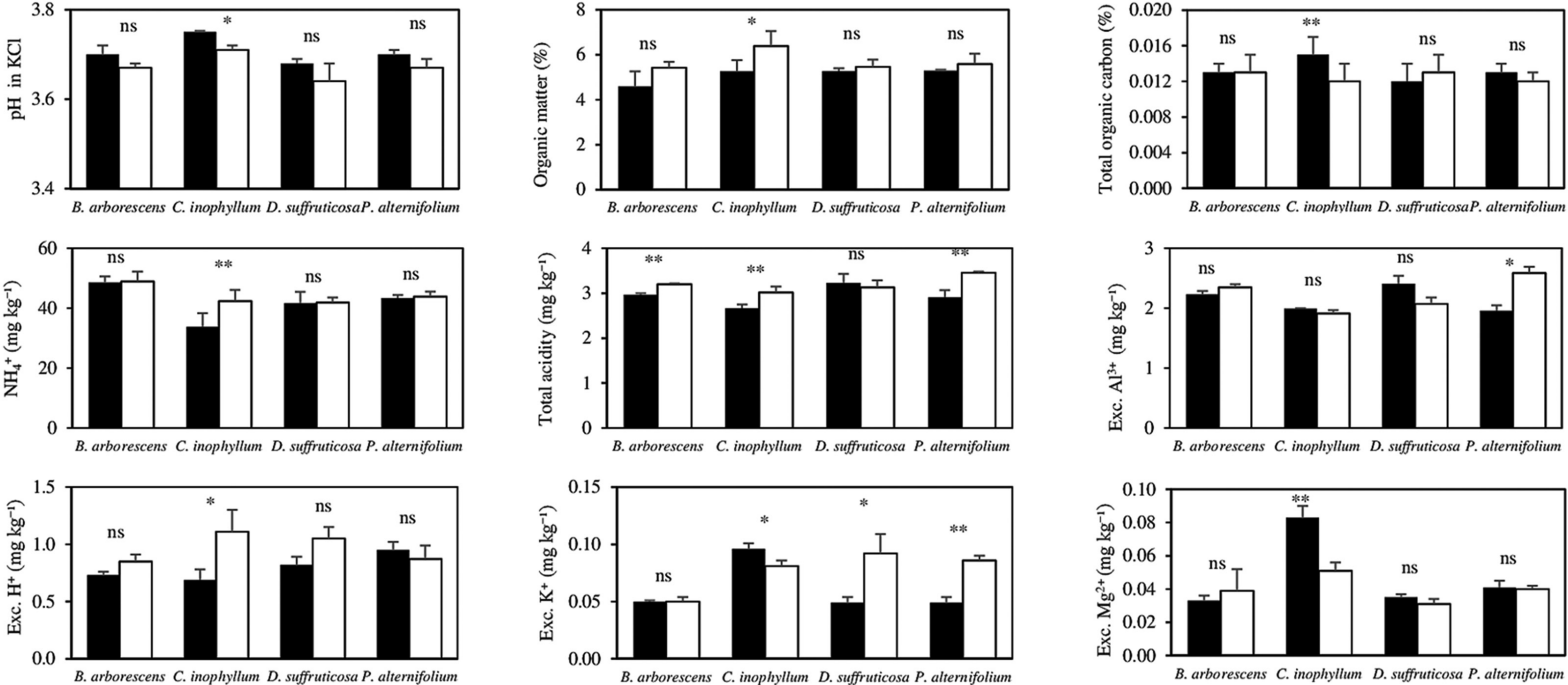
Variations of selected soil chemical properties after incubation with different litter compositions of native species (*B. arborescens*, *D. suffruticosa*, *C. inophyllum* and *P. alternifolium*) in the absence (closed bars; T2, T3, T4 and T5, respectively) and the presence (open bars; T6, T7, T8 and T9, respectively) of *A. mangium* leaf litter. Values are expressed as mean ± standard error, SE for *n* = 3 and significances of mean differences between the absence or presence of *A. mangium* litter (5% significance level) were conducted by *t*-test and expressed as: ns: non-significant, *: *p* < 0.05, **: *p* < 0.01, ***: *p* < 0.001). The results of pH (KCl), exchangeable concentrations of Ca^2+^ and ${\text{NO}}_{3}^{-}$, and available P concentration were excluded because mean differences between the absence or presence of *A. mangium* litter across all native species were not significant.

Soils incubated with litter of native heath forest species showed significant differences for selected treatments in the presence and absence of *A. mangium* litter (*p* < 0.05; Fig. 1). The most substantial significant effects on soil properties were seen for those soil samples incubated with *C. inophyllum* litter in the absence or presence of *A. mangium* litter. Soil samples incubated with *C. inophyllum* litter in the absence of *A. mangium* litter were significantly lower in pH in KCl, organic matter content, concentrations of exchangeable ammonium ( ${\text{NH}}_{4}^{+}$) and H^+^, and total acidity than when incubated in the presence of *A. mangium* litter. Conversely, treatment 3 soil samples were significantly higher in total organic carbon, exchangeable K^+^ and Mg^2+^ content than treatment 7 samples.

For those soil samples incubated with *B. arborescens* litter, only total acidity was significantly lower when compared to those samples incubated with *A. mangium* litter (Fig. 1). For soil samples incubated with litter from the pioneer species, *D. suffruticosa*, only exchangeable K^+^ was significantly lower when compared with those samples incubated with *A. mangium* litter (Fig. 1).

In those soil samples incubated with *P. alternifolium* litter, total acidity was significantly lower when compared to those samples having *A. mangium* litter (Fig. 1). Similarly, exchangeable K^+^ was significantly lower in the absence of *A. mangium* litter (0.049 ± 0.005 mg kg^−1^) compared to those with *A. mangium* litter (0.086 ± 0.004 mg kg^−1^), and exchangeable Al^3+^ was significantly lower when compared to those having *A. mangium* litter (Fig. 1).

## DISCUSSION

### Differences in Mass Litter Loss among Litter Incubation Treatments

The percentage of mass litter loss after 9 months of incubation differed significantly between the invasive *A. mangium* and two of the four native tropical heath forest species investigated (*D. suffruticosa and C. inophyllum*). *Acacia mangium* litter appeared to significantly decompose faster than *D. suffruticosa* litter but slower than *C. inophyllum* litter. In contrast, decomposition of *A. mangium* litter appeared similar to those of *B. arborescens* and *P. alternifolium* litter as there were no significant differences in percentage mass litter loss between these three species.

A similar decomposition pattern was observed for incubation experiments carried out by Castro-Díez *et al*. (2012) to assess exotic species vs. natives in Spain where exotic species leaf litter (*Eucalyptus globulus* and *Acacia dealbata*) decomposed relatively slower than native species leaf litter (*Quercus robur* and *Pinus pinaster*). Our results differ from Yusof (2015) and Suhaili (2017) who reported faster leaf litter decomposition rates for *A. mangium* than native tropical heath forest litter (*Symplocos polyandra*, *Melastoma malabathricum* and *Callophyllum soulatrri*). However, our findings are consistent with Jaafar *et al*. (2022b) who reported no significant differences in leaf litter decomposition rates between *Acacia* and mixed-species for invasive *Acacia* invaded vs. non-invaded heath forests in Brunei Darussalam. These differences between studies can be attributed to differences in study settings (i.e., laboratory controlled conditions for ours and Castro-Díez *et al*. (2012) vs. field conditions for Yusof (2015), Suhaili (2017) and Jaafar *et al*. (2022b), and the selection of different native species co-occurring with invasive acacias.

As our soil incubation experiment was conducted in a laboratory setting under uniform environmental conditions for all samples, the differences in percentage mass litter loss between *A. mangium*, *D. suffruticosa* and *C. inophyllum* could have resulted from differences in their litter quality affecting litter decomposition rates. Our determination of nutrient content of the fresh litter of these species showed that *C. inophyllum* leaves recorded significantly lower total N, total K, total Ca and total Mg content than those found in *A. mangium* phyllodes and *D. suffruticosa* leaves. Higher foliar nutrient content can facilitate decomposition because it provides essential elements that microorganisms need to break down organic matter more efficiently. Interestingly, we found highest soil exchangeable Mg content recorded for Treatment 3 (soils incubated with *C. inophyllum* litter), despite its fresh leaves showing the lowest Mg content among the five species studied. We suggest that *C. inophyllum* litter may have released nutrients faster as it appeared to decompose fastest (i.e., highest mean percentage of mass litter loss of 27.89%). The lowest mean percentage of mass litter loss recorded in *D. suffruticosa* (i.e., 2.20%) may be attributed to the chemical composition of *D. suffruticosa* leaves, such as high lignin or polyphenol content (Wickramathilake *et al*. 2014), as well as the presence of structural components such as cellulose and hemicellulose (Wahyuni 2023), both of which can slow down decomposition rates. Additionally, *A. mangium* phyllodes are xeromorphic with higher secondary metabolites contents such as tannins, flavonoids and alkaloids (Jaafar 2020), both of which can be attributed to the xeromorphic characteristics of its leaves. Xeromorphic adaptations, such as thick cuticles, reduced leaf surface area and dense trichomes, can contribute decreased decomposition rates. This also likely explains the lack of differences in percentage mass loss between *A. mangium*, *B. arborescens* and *P. alternifolium* litter, as these three species have fairly similar xeromorphic leaf characteristics (Yusof 2015). However, further studies using the litter bag technique in both the field and lab setting, including measurements of nutrient release and other liltter decomposition parameters, should be conducted to better understand these initial findings.

### Effects of Incubation with Single-species Litter on Soil Chemical Properties

The effects of incubation with a single-species of native tropical heath forest species litter were variable and species-specific. Out of the four native heath forest species used in our study, soil samples incubated with *C. inophyllum* litter showed variations in pH_water_ and pH_KCl_ and exchangeable concentrations of Al^3+^, H^+^, K^+^, Mg^2+^ and nitrate ( ${\text{NO}}_{3}^{-}$) and total acidity when compared with soils incubated without litter. However, total organic C content, organic matter content, exchangeable NH_4_^+^ concentration and available P concentration were not affected by the leaf litter type.

Soils incubated with *C. inophyllum* litter had significantly higher pH_water_ and pH_KCl_ and were enriched with exchangeable concentrations of ${\text{NO}}_{3}^{-}$, K^+^ and Mg^2+^ but lower in total acidity and exchangeable Al^3+^ concentration compared to soils not incubated with litter. This may be partly due to greater percentage mass litter loss recorded in *C. inophyllum* than the pioneer species (*D. suffruticosa*), native heath species (*P. alternifolium*) and invasive *A. mangium*. Higher mass loss often translates to greater nutrient release, which returns nutrients to the incubated soils (Berg & McClaugherty 2008).

Although fresh *C. inophyllum* leaves recorded lower leaf nutrient contents for total N, Ca and Mg (Table 2), it appeared that after the 9-months incubation experiment, decomposing *C. inophyllum* litter increased cation (K^+^ and Mg^2+^) content and nitrates, thus resulting in lower soil acidity. It is likely that the higher ${\text{NO}}_{3}^{-}$ concentration in soil after incubation with *C. inophyllum* resulted from the oxidation of organic nitrogen (total N) to inorganic nitrogen ( ${\text{NO}}_{3}^{-}$) during decomposition (Marchante *et al*. 2009). Joshi *et al*. (2019) reported similar findings, where the native tree species *Euterpe oleracea* showed a higher decomposition rate that induced the immobilising process where it increased pH and ${\text{NO}}_{3}^{-}$ in the deciduous forest soils in the tropical forests of the Brazilian Amazon.

Results for lower total acidity and exchangeable Al^3+^ content for soils incubated with *C. inophyllum* leaf litter could be linked to the increased levels of pH (both pH_water_ and pH_KCl_). The increased pH values potentially indicate that the presence of *C. inophyllum* leaf litter can remediate the highly acidic tropical heath forest soil by increasing ${\text{NO}}_{3}^{-}$ and cations such as K^+^ and Mg^2+^, present in these habitats. Once the total acidity in soils is lowered, proton ion charges can freely move, encouraging more cation exchange capacity (CEC) to occur in the soils (Bergaya *et al*. 2006) and lessening the stressed soil conditions (Li *et al*. 2001). Higher CEC results in an increase in cations, such as K^+^, Ca^2+^ and Mg^2+^ in the soils, which equates to an increase in soil fertility (Oorts *et al*. 2003).

When comparing the effects of soil incubation with *A. mangium* litter and without any litter, *A. mangium* caused an increase in pH_water_ and exchangeable ${\text{NO}}_{3}^{-}$ and K^+^ concentrations in the heath soils but decreased total acidity and exchangeable Al^3+^ concentration in the incubated soils without litter. Higher N and P content of *A. mangium* litter could be a reason for this pattern as nitrogen produces nitrates ( ${\text{NO}}_{3}^{-}$) and releases hydrogen H^+^ ions during the nitrification process (Marchante *et al*. 2009; Pandey *et al*. 2000). The resulting H^+^ ions are then used in P mineralisation or specifically for the formation of orthophosphates, ${\text{H}}_{2}{\text{PO}}_{4}^{-}$ (Barea & Richardson 2015). The binding of hydrogen with phosphate requires at least two H ions, which results in an increase in soil pH but a decrease in total acidity and exchangeable Al^3+^ concentration in the incubated soils.

Soils incubated with *B. arborescens* litter recorded significantly higher pH in water and exchangeable K^+^ concentration, but lower exchangeable Al^3+^ concentration than the soils without litter. Soils incubated with *D. suffruticosa* litter recorded significantly higher exchangeable K^+^ and Mg^2+^ concentrations than soils without litter. Meanwhile, soils incubated with *P. alternifolium* recorded significantly higher exchangeable H^+^, ${\text{NO}}_{3}^{-}$, Mg^2+^ and Ca^2+^ concentrations, but lower total acidity and exchangeable Al^3+^ concentrations. These results show a trend that the nutrient contents may be positively associated with percentage mass litter loss. We suggest that species with lower percentage litter loss may be less effective in altering incubated soil properties. Our results are comparable with Turner *et al*. (2000) that reported species with thicker leaves are low in foliar nitrogen and ash concentrations.

### Effects of Incubation with a Single-species Litter in the Presence or Absence of *A. mangium* Litter on Soil Chemical Properties

When comparing the effects of incubation with each of the four different tropical heath forest species litter in the presence of *A. mangium* leaf litter, it was identified that soils incubated with a combination of *C. inophyllum* and *A. mangium* leaf litters recorded the highest number of significant effects in terms of increasing pH_water_, OM content, total acidity and exchangeable concentrations of ${\text{NH}}_{4}^{+}$ and H^+^, and decreasing TOC and exchangeable concentrations of K^+^ and Mg^2+^. This is consistent with the results of the parallel single-species litter treatments that also recorded the most significant effects on soil samples when incubated with *C. inophyllum* litter alone and *A. mangium* litter alone. Furthermore, the presence of *A. mangium* litter with *C. inophyllum* litter enriched the overall nutrient concentration compared to *A. mangium* litter alone by increasing OM, ${\text{NH}}_{4}^{+}$ after the incubation period. This can be attributed to the presence of high contents of nitrogen in *A. mangium* litter. Yusof (2015) also reported a similar positive correlation between leaf litter N and the topsoil N content in an *Acacia*-invaded tropical heath forest in Borneo.

In contrast, when tropical heath forest soils were incubated with litter of the other three native species in combination with *A. mangium* litter, significant changes were only recorded for total acidity (*B. arborescens* and *P. alternifolium*), exchangeable Al^3+^ (*P. alternifolium*) and K^+^ (*D. suffruticosa* and *P. alternifolium*) concentrations. Based on these findings, we suggest that the native *C. inophyllum* appears to be a promising candidate for enrichment planting of degraded or disturbed tropical heath forests with co-occurring invasive *A. mangium*. The presence of *C. inophyllum* litter appeared to lessen impacts of *A. mangium* through decreased soil acidity and increased organic matter and macronutrients (e.g., ${\text{N-NH}}_{4}^{+}$) in the heath soils. Enrichment planting of *C. inophyllum* in *Acacia-*invaded heath forests, as well as degraded areas, in Brunei and elsewhere in Borneo could therefore facilitate the recovery of invaded and degraded soils as a first step towards the restoration of these ecosystems.

The findings of this study imply that other three tropical heath forest species litter, in the presence of *A. mangium* leaf litter (mixed species litter incubation), can also potentially improve most of the soil chemical properties, such as pH, OM, total acidity and exchangeable concentrations of ${\text{NH}}_{4}^{+}$, H^+^, Al^3+^ and K^+^, when compared to incubation with *A. mangium* litter alone. The negative impacts shown by *A. mangium* leaf litter can be due to the presence of higher lignin, TPC (Total phenolic content) and TTC (Total tannin content) present in phyllodes (Jamil 2018), that could hinder the decomposition rate. Turner *et al*. (2000) and Yusof (2015) also reported that tropical heath forest species had significantly thicker leaves and were higher in foliar nitrogen and ash concentrations. The effects of native litter in combination with the invasive *A. mangium* litter on soil properties appear to be species-specific, possibly due to differences in litter decomposition rates among the native species, as well as differences in leaf litter quality of these native species. Based on the results of percentage of litter loss, it shows that decomposition of *C. inophyllum* litter is higher compared to the other three native species and *A. mangium* litter; thus, implying significant improvements on soil properties when incubated with *C. inophyllum* litter.

The presence of *A. mangium* leaf litter increased total acidity in tropical heath forest soils investigated, thus causing a negative impact. The increase in total soil acidity with the presence of *A. mangium* leaf litter may be due to the release of humic acid from *A. mangium* leaf litter (Valladares *et al*. 2016). Phyllodes of *A. mangium* contain high levels of N and P (Yusof 2015) and litter containing higher levels of N and P are known to release more humic acids (Anđelković *et al*. 2006). It is possible that continuous exposure of tropical heath forest soils to *A. mangium* leaf litter, especially in high volumes in leaf litterfall (Suhaili 2017; Jaafar *et al*. 2022b), may also trigger other negative effects such as nutrient leaching and Al toxicity. Our findings have shown that the incorporation of native species leaf litter through mixed species incubation can mitigate these negative effects, providing a stabilising effect on soil pH and nutrient balance, thereby potentially restoring the health and resilience of invaded tropical heath forest soil properties.

### Management Implications

Our results offer important insights for Bornean tropical heath forests impacted by *A. mangium*. Long-term invasions of *A. mangium* in Bornean heath forests existing on nutrient-poor and highly acidic soils, when left unmanaged, are known to drastically modify soil properties (Jaafar *et al*. 2022b, Ibrahim *et al*. 2023) leading to changes in ecosystem functioning that prevent natural ecosystem recovery. The recorded variable effects on soil chemical properties when incubated with *A. mangium* and different single-species litter of four native tropical heath forest species suggests that a uniform strategy for the management of *A. mangium* invasion may not be effective as different native species likely interact with *A. mangium* in complex and distinct ways. Further studies, conducted over longer periods than the duration of our study (nine months), focusing on native species that co-occur with *A. mangium* can help forest managers make informed decisions to formulate effective management and mitigation strategies.

## CONCLUSION

Our soil incubation experiment has demonstrated that most of the measured chemical properties of tropical heath forest soil samples incubated with *A. mangium* leaf litter were significantly altered. Conversely, the effects on soil chemical properties when incubated with a single native tropical heath forest species litter did not show any consistent patterns and appeared to be species-specific. The effects on soil properties when each of the four tropical heath forest species litter were incubated with *A. mangium* litter varied, with the most desirable impacts recorded when incubated with *C. inophyllum* litter (increased pH_water_, OM content, total acidity and exchangeable concentrations of ${\text{NH}}_{4}^{+}$ and H^+^, and decreased TOC and exchangeable concentrations of K^+^ and Mg^2+^). These positive impacts of *C. inophyllum* may indicate that it is a promising candidate for enrichment planting of degraded or disturbed tropical heath forests with co-occurring invasive *A. mangium*.

## Figures and Tables

**Table 1:** Selected leaf nutrient contents (*n* = 3) of the leaves of four native tropical heath forest species (*B. arborescens*, *C. inophyllum*, *D. suffruticosa* and *P. alternifolium)* and one invasive tree species, *A. mangium* before incubation experiment

Species	Leaf nutrient contents				

	Total N (mg/g)	Total P (mg/g)	Total K (mg/g)	Total Ca (mg/g)	Total Mg (mg/g)
*A. mangium*	17.17 ± 0.32^a^	0.83 ± 0.01^a^	6.53 ± 0.04^b^	39.86 ± 0.92^b^	2.96 ± 0.76^a^
*B. arborescens*	17.88 ± 0.23^a^	0.59 ± 0.03^b^	3.49 ± 0.01^e^	32.07 ± 0.29^c^	4.31 ± 0.29^a^
*C. inophyllum*	9.97 ± 0.32^c^	0.45 ± 0.02^c^	6.07 ± 0.04^c^	21.47 ± 0.71^d^	2.42 ± 0.14^a^
*D. suffruticosa*	13.75 ± 0.21^b^	0.04 ± 0.00^e^	10.63 ± 0.04^a^	40.87 ± 0.22^b^	2.72 ± 0.14^a^
*P. alternifolium*	13.64 ± 0.16^b^	0.35 ± 0.01^d^	5.45 ± 0.07^d^	44.37 ± 0.71^a^	3.72 ± 0.08^a^

*Notes*: Data are expressed as mean ± standard error, SE and significances of between-treatments differences (95% confidence levels or *p* < 0.05) were conducted by Tukey test. Means in same column with different letters are significantly different (*p* ≤ 0.05).

**Table 2:** Treatments of tropical heath forest soils incubated with leaf litter of four native species (*B. arborescens, C. inophyllum, D. suffruticosa* and *P. alternifolium*) and the invasive *A. mangium*.

Treatment #	Leaf weight of species investigated
T0	0 g leaves (soil column with no litter)
T1	24 g of *A. mangium*
T2	24 g of *B. arborescens*
T3	24 g of *C. inophyllum*
T4	24 g of *D. suffruticosa*
T5	24 g of *P. alternifolium*
T6	12 g of *A. mangium* + 12 g of *B. arborescens*
T7	12 g of *A. mangium* + 12 g of *C. inophyllum*
T8	12 g of *A. mangium* + 12 g of *D. suffruticosa*
T9	12 g of *A. mangium* + 12 g of *P. alternifolium*

*Note*: Total treatment = 10, number of replications per treatment = 3

**Table 3:** Percentage mass litter loss and selected soil chemical variables for tropical heath forest soils incubated without any litter (T0) and with a single-species litter of *A. mangium* litter (T1) and selected native species (*B. arborescens* – T2, *C. inophyllum* – T3, *D. suffruticosa* – T4 and *P. alternifolium* – T5).

Variable	Treatment					

	Control (T0)	*A. mangium* (T1)	*B. arborescens* (T2)	*C. inophyllum* (T3)	*D. suffruticosa* (T4)	*P. alternifolium* (T5)
Mass litter loss (%)	NA	16.54 ± 1.08^b^	19.97 ± 3.39^ab^	27.89 ± 0.78^a^	2.20 ± 0.75^c^	15.36 ± 1.40^b^
pH _in KCI_	3.65 ± 0.003^b^	3.72 ± 0.024^ab^	3.70 ± 0.020^ab^	3.75 ± 0.003^a^	3.68 ± 0.010^ab^	3.70 ± 0.010^ab^
pH _in water_	4.61 ± 0.031^c^	4.94 ± 0.056^a^	4.86 ± 0.034^a^	4.83 ± 0.071^ab^	4.63 ± 0.032^bc^	4.79 ± 0.033^abc^
${\text{NO}}_{3}^{-}$ (mg kg ^1^)	3.47 ± 0.073^c^	3.92 ± 0.093^ab^	3.72 ± 0.067^abc^	3.98 ± 0.017^a^	3.58 ± 0.142^bc^	3.90 ± 0.029^ab^
Total acidity (mg kg^−1^)	3.24 ± 0.064^a^	2.88 ± 0.023^c^	2.97 ± 0.018^abc^	2.67 ± 0.047^c^	3.23 ± 0.114^ab^	2.91 ± 0.093^bc^
Exchangeable Al^3+^ (mg kg^−1^)	2.55 ± 0.029^a^	2.20 ± 0.031^c^	2.23 ± 0.033^bc^	1.99 ± 0.006^d^	2.41 ± 0.074^ab^	1.96 ± 0.052^d^
Exchangeable H^+^ (mg kg^−1^)	0.69 ± 0.037^b^	0.68 ± 0.012^b^	0.73 ± 0.018^b^	0.69 ± 0.052^b^	0.82 ± 0.040^ab^	0.95 ± 0.041^a^
Exchangeable K^+^ (mg kg^−1^)	0.038 ± 0.0008^d^	0.072 ± 0.0023^b^	0.050 ± 0.0005^c^	0.096 ± 0.0031^a^	0.049 ± 0.0025^c^	0.049 ± 0.0026^cd^
Exchangeable Ca^2+^ (mg kg^−1^)	0.013 ± 0.0003^b^	0.026 ± 0.0019^b^	0.016 ± 0.0002^b^	0.023 ± 0.0007^b^	0.028 ± 0.0006^b^	0.073 ± 0.006^a^
Exchangeable Mg^2+^ (mg kg^−1^)	0.022 ± 0.0015^c^	0.032 ± 0.0026^bc^	0.033 ± 0.0015^bc^	0.083 ± 0.0038^a^	0.035 ± 0.0012^b^	0.041 ± 0.0027^b^

*Notes*: Values are expressed as mean ± standard error, SE for *n* = 3 and significances of between-treatments differences (5% significance levels or *p* < 0.05) are conducted by Tukey’s test after a one-way ANOVA. Means with different letters within the same row are significantly different (*p* < 0.05). No significant differences between treatments were detected for total organic carbon (TOC), organic matter (OM), exchangeable ${\text{NH}}_{4}^{+}$ concentration and available P concentration and these variables were not presented here.
